# Mapping study of papillary thyroid carcinoma in China: Predicting EQ-5D-5L utility values from FACT-H&N

**DOI:** 10.3389/fpubh.2023.1076879

**Published:** 2023-02-23

**Authors:** Deyu Huang, Jialing Peng, Na Chen, Qing Yang, Longlin Jiang

**Affiliations:** ^1^School of Nursing, Chengdu Medical College, Chengdu, China; ^2^Sichuan Cancer Hospital and Institute, Sichuan Cancer Center, School of Medicine, University of Electronic Science and Technology of China, Chengdu, China

**Keywords:** papillary thyroid carcinoma, mapping, FACT-H&N, EQ-5D-5L, Beta mixture regression model

## Abstract

**Objective:**

To develop a mapping algorithm that can be used to predict EQ-5D-5L health utility scores from FACT-H&N and obtain health utility parameters for Chinese patients with papillary thyroid carcinoma (PTC), which can be used for cost-utility analysis in health economic.

**Methods:**

A total of 1,050 patients with PTC from a tertiary hospital in China were included, and they completed FACT-H&N and EQ-5D-5L. Four mapping algorithms of direct mapping functions were used to derive the models: Ordinary least squares (OLS), Tobit model (Tobit), Two-part model (TPM), and Beta mixture regression model (Beta). The goodness-of-fit of models was assessed by the mean absolute error (MAE), root mean square error (RMSE), Akaike information criteria (AIC), Bayesian information criteria (BIC), and absolute error (AE). A fivefold cross-validation method was used to test the stability of the models.

**Results:**

The mean utility value of the EQ-5D-5L was 0.870 ± 0.094. The mean EQ-VAS score was 76.5 ± 13.0. The Beta mixture regression model mapping FACT-H&N to EQ-5D-5L achieved the best performance [fivefold cross-validation MAE = 0.04612, RMSE = 0.06829, AIC = −2480.538, BIC = −2381.137, AE > 0.05 (%) = 32.48, AE > 0.1 (%) = 8.95]. The independent variables in this model were Physical Well-Being (PWB), Emotional Well-Being (EWB), Head & Neck Cancer Subscale (HNCS) scores and its square term and interaction term scores.

**Conclusions:**

This study calculated the health utility score of Chinese patients with PTC. The reported algorithms can be used to map the FACT-H&N into the EQ-5D-5L, which can be applied in the cost-utility related study of patients with PTC.

## 1. Introduction

Thyroid cancer is a most common head and neck cancer, with papillary thyroid carcinoma (PTC) accounting for approximately 90% in thyroid cancer (1). According to the global cancer statistics (2), the thyroid cancer is the ninth most malignant tumor. In China, the incidence and mortality of thyroid cancer continue to rise (3), affecting the patient's health-related quality of life (HRQoL) and the utilization of health resources. Health economic evaluation plays a key role in medical resource allocation and clinical decision-making, and its preferred method is cost-utility analysis (4).

In the cost-utility analysis of cancer treatment, the most important and commonly used health outcomes are quality-adjusted life years (QALYs) (5, 6). Calculating QALYs requires measuring health utility values. The EuroQol five-dimensional 5 level (EQ-5D-5L) is currently the most widely used preference-based health utility instrument at home and abroad that can be used to calculate health utility values (7, 8). Functional Assessment of Cancer Therapy (FACT) is one of the most widely used instruments to measure health-related quality of life in cancer patients (9) and related mapping studies have covered breast cancer (10–13), ovarian cancer (14), colorectal cancer (15, 16), lung cancer (15, 17), prostate cancer (18, 19) and others. However, FACT-H&N (Functional assessment questionnaire for the treatment of head and neck cancer) is a non-preference-based instrument and cannot be directly used for calculating health utility values. Meanwhile, in clinical experiments or work, the EQ-5D-5L is not universally measured, and non-preference-based instruments are more widely used. In such cases, it is common to use “mapping” to convert the available health status data from a non-preference-based measure to utility values for a generic preference-based measure (20). The mapping methods include direct mapping and indirect mapping methods (21). In this study, we adopted direct mapping method.

Ordinary least squares (OLS), Tobit model (Tobit), and Two-part model (TPM) are the most common mapping models of direct mapping functions, and Beta mixture regression models have been developed and gradually applied in recent years. By reviewing the literature, we did not find mapping studies of the FACT-H&N in thyroid cancer. The purpose of this study was to develop a mapping algorithm from the FACT-H&N to the EQ-5D-5L based on the Chinese PTC population.

## 2. Materials and methods

### 2.1. Study subjects

The study was conducted from May to December 2021 at Sichuan Cancer Hospital, a large tertiary-grade oncology hospital that provides medical services to most cancer patients in southwestern China. The inclusion criteria for this study were as follows: (1) patients with PTC diagnosed by pathology; (2) aged between 18 and 80 years old; (3) clear thinking, normal spirit, and a certain ability to understand and express; and (4) willing to participate in this study and sign the “informed consent form”. A total of 1,100 patients with PTC participated in the survey, and some patients were excluded due to missing values in their survey. Thus, 1,050 patients who completed the entire questionnaires were included in the data analysis. Three investigators who had received strict training participated in the investigation. The questionnaires included the general demographic data of the patients and instruments (FACT-H&N, EQ-5D-5L). The clinical treatment information of the patients was provided by the electronic medical record system of the hospital. After all questionnaires were completed, three investigators jointly checked whether there were any missing items so that we could contact the respondents.

### 2.2. Instruments

#### 2.2.1. FACT-H&N

The FACT-H&N was designed at the Rush University Medical Center in Chicago, USA. The Chinese version of the FACT-H&N has good reliability and construct validity and can be used to determine the quality of life of Chinese patients with head and neck cancer (22–24). We contacted the research institution of the FACT-H&N; obtained the Chinese version and the scoring rules; and obtained the authorization of the instrument. The FACT-H&N investigates the situation of patients seven days before the day of investigation, and the specific subscale include Physical Well-being (PWB), Social/Family Well-being (SWB), Emotional Well-being (EWB), Functional Well-being (FWB) and Head & Neck Cancer Subscale (HNCS), with a total of 39 items. The scores of the items contained in each domain were summed to obtain the crude score of this domain, and the scores of these five domains were summed to obtain the total score, which ranged from 0 to 148, where a higher overall score indicates a better corresponding quality of life (25, 26).

#### 2.2.2. EQ-5D-5L

The EQ-5D-5L was developed by the European Quality of Life Group, which is a commonly used preference-based health utility instrument (27–30). The instrument consists of a five-dimensional self-assessment and visual analogue instrument (EQ-VAS). Five dimensions include: mobility, self-care, daily activities, pain or discomfort, anxiety and depression, and each dimension is divided into no, slight, moderate, severe, extreme problems. The EQ-VAS is a instrument marked with numbers from zero to one hundred, with one hundred representing the best imaginary health condition and zero representing the worst imaginable health condition. The patients mark the instrument according to their perceived health condition on the day of the survey. We contacted the research and development institution of the EQ-5D-5L and obtained the instrument authorization and scoring rules. We used the Chinese population tariff to calculate the health utility score (31, 32), which can provide a valid reference for this value set. The Chinese tariff of the EQ-5D-5L was developed by the time trade-off (TTO) technique, which has a theoretical range of scores from −0.391 to 1.0 (33).

### 2.3. Statistical analysis

#### 2.3.1. Descriptive statistical analysis

Statistical measures such as percentage, mean, and standard deviation were used to describe the patient characteristics. In our study, EQ-5D-5L health utility data showed a skewed distribution; therefore, univariate analysis was performed using the rank sum test for patient characteristics to obtain factors affecting health utility, of which the Wilcoxon rank sum test was used for two-category data and the Kruskal–Wallis *H*-test was used for multicategory data. Multivariate analysis of factors influencing health utility values using Tobit regression.

#### 2.3.2. Correlations between instruments

Spearman's correlation coefficient was used to explore the correlation between the EQ-5D-5L and the FACT-H&N, if the correlation is poor, then the mapping function will perform poorly (34). The reference value for the strength of the relationship are as follows: 0–0.19 is regarded as very weak, 0.2–0.39 as weak, 0.40–0.59 as moderate, 0.6–0.79 as strong and 0.8–1 as very strong correlation (35).

#### 2.3.3. Establishment of the mapping model

The following four modeling approaches were used to predict EQ-5D-5L health utility values.OLS is the most widely used method in the mapping literature (36, 37) and is a commonly used linear regression model. However, OLS can be affected by the ceiling effect, where a bias of underestimation with high values and overestimation with low values will occur; thus, the model is theoretically not fully applicable to the mapping of health utility values (38).

In some cases, Tobit model can mitigate ceiling effects in health utility measures. Tobit is a censored model that aims to estimate the linear relationship between variables when the dependent variable is left-censored or right-censored (39). Tobit is sensitive to violations of heteroskedasticity or non-normality (40).

The TPM model is considered an alternative estimation ways when analyzing skewed data, which reduces the bias in highly skewed distributions caused by the ceiling effect of the utility score (41). The first part of the TPM model uses logistic regression to estimate the probability that a patient is perfectly healthy, and the second part uses the previous OLS model to estimate a patient's health utility score when not perfectly healthy and then combines the two models to obtain the final health utility value.

Because mapping model's predictions can be outside the bounds, the typical linear regression models do not fit a bounded dependent variable and cannot manage extreme values (zero and one) on the bounds of that interval. Beta mixture regression models provide flexible means to regress outcome distributions with truncation support. The disadvantage of this model is that the irregularity of the distribution leads to inconsistent parameter estimates. The model generalizes to values that allow either or both boundaries by adding a degenerate distribution with a probability mass at the boundaries, which is called the truncated-inflated Beta model. The Beta model is flexible, where the first part analyses an incompletely healthy sample, and the second part uses the full sample and, based on the first part, estimates an inflated truncated mixed beta regression. The Beta model can fit models with and without truncation, as well as truncate models at the bottom or top of an interval range. It can predict health tools at various points, including negative values, observed peaks at full health or death, gap values between boundaries, and a mixture of the numeric components of the beta distribution. The command “truncation” in Stata software is used to determine whether there is a truncation in the model (42). Here we only consider the inflation part of the model at perfect health.

In our study, the mapping models of OLS, Tobit, TPM, and Beta were used to evaluate the following six different sets of independent variables:

Mode l: FACT-H&N total score as the primary predictor of health utility score, such as OLS1, Tobit1, etc.

Model 2: Such as OLS2, Tobit2, etc. The independent variables of the models were the score of each subscale of the FACT-H&N, including PWB, SWB, EWB, FWB, HNCS.

Model 3: Such as OLS3, Tobit3, etc. The independent variables were the meaningful subscale scores in each dimension score of Model 2: PWB, EWB, HNCS, where *P* < 0.01 were considered significant.

Model 4: Such as OLS4, etc. The independent variables were those of Model 3 and the squared terms of the meaningful subscale scores (PWB, EWB, HNCS).

Model 5: Such as OLS5, etc. The independent variables were those of Model 4 and the interaction terms of the meaningful subscale scores (PWB, EWB, HNCS).

Model 6: Such as OLS6, etc. The independent variables were those of Model 5 and age, gender.

The Beta model was explained here (see **Table 3** for details):

Beta 1a: the first model of Beta was the total score of FACT-H&N, and there was one component.

Beta 1b: the first model of Beta was the total score of FACT-H&N, and there were two components.

Beta 1c: the first model of Beta was the total score of FACT-H&N, and there were three components.

Beta 2a: the first model of Beta was each dimension of the instrument, and there was one component.

Beta 3a: the first model of Beta was a meaningful dimension, there was one component, etc. Because the Beta model may have problems with convergence, some models can only have one component.

Beta model without truncation: the Beta model without a cut-off value.

Beta with truncation: the Beta model with a cut-off value.

#### 2.3.4. Model validation and evaluation

A fivefold cross-validation method was used to test the predictive performance of the model, and all samples were randomly divided into two groups: 80% of the samples were used to estimate the dataset, 20% of the samples were used to validate the dataset, and the above four mapping algorithms were used to predict health utility values. The prediction procedure was repeated five times, and the mean absolute error (MAE), root mean square error (RMSE), Akaike information criteria (AIC), Bayesian information criteria (BIC), and absolute error (AE) of the nine better models were obtained. The smaller the values were, the better the model performance. Values from these indicators were ranked and summed to generate an average ranking (ARV). The model with the lowest ARV would be chosen as the optimal model (43, 44). In addition, the performance of the model can also be visualized by plotting the scatter plot, error histogram, etc. All statistical analyses were performed in Stata version 16.0.

## 3. Results

### 3.1. Patient characteristics and descriptive analysis

A total of 1,050 patients with PTC were included in this study. Women account for 76% of the sample size. The average age of the overall sample was 40.76 years old. Table 1 showed the patient characteristics. The mean utility score on the EQ-5D-5L was 0.870 (standard deviation 0.094), and the mean on the EQ-VAS was 76.5 (standard deviation 13.0). The mean of the FACT-H&N was 108.152 (standard deviation 15.478). The highest score in each subscale of the FACT-H&N was EWB, and the lowest score was FWB. Among the dimensions of the EQ-5D-5L, the dimension that accounted for the largest proportion was no difficulty in mobility (90%).

**Table 1:** Patient characteristics (*n* = 1,050).

**Variables**	**Grouping**	**Number of people**	**Composition ratio (%)**
Gender	Male	252	24.00
	Female	798	76.00
Age at time of survey	18 − 44	642	61.14
	45 − 54	267	25.43
	55 − 64	117	11.14
	65 − 80	24	2.29
Educational attainment	Literacy and primary school	90	8.57
	Junior high school	172	16.38
	Senior high school	181	17.24
	College	242	23.05
	Undergraduate	323	30.76
	Master's degree or above	42	4.00
Marital status	Unmarried	150	14.29
	Married	855	81.43
	Divorced	30	2.86
	Widowed	9	0.86
	Others	6	0.57
Occupation	Civil servants/public institutions/company employees	465	44.29
	Farmer/worker	167	15.90
	Self-employed/Freelance	117	11.14
	Unemployed	46	4.38
	Retired	74	7.05
	Others	181	17.24
Monthly household income	<2000 RMB	67	6.38
	2000–4999 RMB	310	29.52
	5000–9999 RMB	403	38.38
	10000–29999 RMB	223	21.24
	≥30000 RMB	47	4.48
TNM stage	I	984	93.71
	II	57	5.43
	III	5	0.48
	IV	4	0.38
Current treatment	Surgery	700	66.67
	Iodine 131	100	9.52
	Medicine treatment	250	23.81

### 3.2. Correlations between instruments

**Figure 1:**
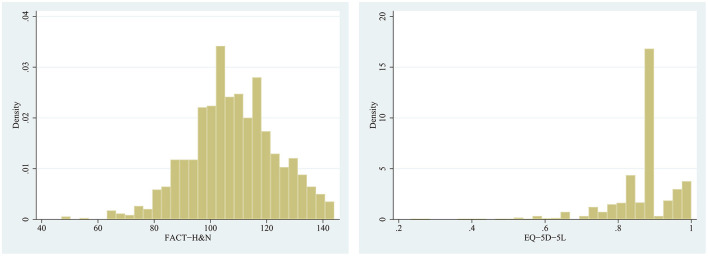
Histograms of the FACT-H&N and the EQ-5D-5L scores.

Figure 1 showed histograms of the FACT-H&N and the EQ-5D-5L scores, with the data for both scales being right skewed. Table 2 Spearman rank correlation analysis showed that the total score of EQ-5D-5L was significantly positively correlated with the total score of FACT-H&N (Spearman correlation coefficient was 0.621). The total score of the EQ-5D-5L and the scores of each dimension of the FACT-H&N were correlated, and the correlation coefficient ranged from 0.140 to 0.626. The correlation coefficient between the total score of the FACT-H&N and the scores of each item of the EQ-5D-5L was between −0.497 and −0.243, and the correlation coefficient between the scores of each dimension of the FACT-H&N and the scores of each item of the EQ-5D-5L was −0.704 to 0.039, except for SWB and individual items of HNCS (^*^P < 0.001).

**Table 2:** Correlation coefficient between EQ-5D-5L and FACT-H&N scale scores.

**Variable**	**PWB**	**SWB**	**EWB**	**FWB**	**HNCS**	**FACT-H&N total score**
Mobility	−0.297^*^	−0.097	−0.197^*^	−0.234^*^	−0.267^*^	−0.306^*^
Self-care	−0.338^*^	0.039	−0.261^*^	−0.295^*^	−0.704^*^	−0.486^*^
Daily activities	−0.313^*^	−0.015	−0.220^*^	−0.267^*^	−0.369^*^	−0.358^*^
Pain or discomfort	−0.510^*^	−0.058	−0.337^*^	−0.345^*^	−0.496^*^	−0.497^*^
Anxiety or depression	−0.344^*^	−0.167^*^	−0.322^*^	−0.261^*^	0.004	−0.243^*^
EQ-5D-5L total score	0.626^*^	0.140^*^	0.478^*^	0.489^*^	0.521^*^	0.621^*^

* P < 0.001.

### 3.3. Performance of the model

We used four mapping algorithms to build 32 models, including OLS (OLS1-6), Tobit (Tobit1-6), TPM (TPM1-6), Beta (Beta1a, Beta1b, Beta1c, Beta2a, Beta3a, Beta4a, Beta5a, Beta6a without/with truncation point). The detailed performance of 32 models was shown in Table 3, and we performed an average ranking (ARV) of the performance indicators of each model. The regression coefficients of each model and covariance matrix of coefficients of preferred Beta model were shown in Supplementary Tables 1–5.

**Table 3:** Model performance of four methods.

**Model**	**MAE**	**RMSE**	**AIC**	**BIC**	**AE>0.05(%)**	**AE>0.1(%)**	**ARV**
**OLS**							
OLS1	0.04958	0.07354	−2496.935	−2487.022	35.24	10.38	6.00
OLS2	0.04706	0.06851	−2637.802	−2608.062	33.62	9.71	4.33
OLS3	0.04702	0.06855	−2640.647	−2620.820	33.90	9.52	3.83
OLS4	0.04671	0.06825	−2643.916	−2609.220	32.76	8.76	2.58
**OLS5**	0.04632	0.06781	−2651.481	−2601.915	32.10	8.76	**1.83**
**OLS6**	0.04632	0.06778	−2648.248	−2588.77	32.29	8.86	**2.42**
**Tobit**							
Tobit1	0.05019	0.07416	−1949.883	−1935.014	35.90	11.71	6.00
Tobit2	0.04776	0.06913	−2087.080	−2052.385	34.10	9.24	3.42
Tobit3	0.04781	0.06916	−2090.057	−2065.274	33.71	9.52	3.08
Tobit4	0.04794	0.06924	−2089.579	−2049.926	34.48	9.14	3.92
**Tobit5**	0.04760	0.06886	−2097.729	−2043.207	33.71	9.24	**2.33**
**Tobit6**	0.04757	0.06884	−2094.294	−2029.859	33.81	9.14	**2.25**
**TPM**							
TPM1	0.05074	0.07407	−2237.644	−2227.933	37.62	10.57	5.83
**TPM2**	0.04774	0.06900	−2347.581	−2318.449	34.19	9.43	**2.92**
**TPM3**	0.04773	0.06903	−2351.244	−2331.823	34.67	9.43	**2.58**
TPM4	0.04809	0.06959	−2367.337	−2333.349	37.81	9.05	3.58
**TPM5**	0.04796	0.06925	−2374.835	−2326.280	37.43	9.14	**2.92**
TPM6	0.04800	0.06924	−2372.079	−2313.814	37.43	9.05	3.17
**Beta without truncation**							
Beta1a	0.04933	0.07365	−2938.943	−2914.160	47.14	11.24	11.50
Beta1b	0.04878	0.07370	−3142.604	−3097.995	47.52	10.86	8.17
Beta1c	0.04884	0.07356	−3157.691	−3093.256	47.62	11.14	8.58
Beta2a	0.04614	0.06840	−3098.199	−3033.764	48.38	9.62	6.33
Beta3a	0.04626	0.06840	−3098.493	−3053.884	48.86	9.43	6.08
Beta4a	0.04606	0.06828	−3095.662	−3021.313	49.05	9.14	6.08
**Beta5a**	0.04542	0.06764	−3103.895	−2999.807	49.05	9.33	**5.33**
Beta6a	0.04545	0.06761	−3097.357	−2973.444	49.14	9.33	6.25
**Beta with truncation**							
Beta1a	0.04908	0.07372	−3044.025	−3019.242	49.43	11.14	12.25
**Beta2a**	0.04584	0.06865	−3191.214	−3126.779	49.24	10.00	**5.33**
Beta3a	0.04644	0.06905	−3177.016	−3142.320	49.90	9.90	7.50
Beta4a	0.04651	0.06878	−3182.092	−3127.570	50.19	9.62	6.92
Beta5a	0.04637	0.06869	−3185.826	−3121.391	50.29	9.71	7.08
Beta6a	0.04634	0.06867	−3179.198	−3094.937	50.29	9.81	7.58

The bold values indicates the two best-performing models for each approach.

In each model, we selected the two best models (the two lowest ARV models) for final model screening. OLS5 and OLS6, Tobit5 and Tobit6, TPM2, TPM3, and TPM5, Beta 5a without truncation and Beta 2a with truncation models performed best in their respective mapping algorithms. Fivefold cross-validation was performed on the selected nine models, as shown in Table 4. After comprehensive consideration, the EQ-5D-5L utility scores was best predicted by the Beta 5a (without truncation point) consisting of PWB scores, EWB scores, HNCS scores and its square and interaction terms scores (there was no cutoff value, component was 1).

**Table 4:** Five-fold cross-validation of the best-fitting model.

**Model**	**MAE**	**RMSE**	**AIC**	**BIC**	**AE > 0.05 (%)**	**AE > 0.1 (%)**	**ARV**
OLS5	0.04701	0.06828	−2121.049	−2073.715	32.86	8.86	2.67
OLS6	0.04732	0.06895	−2119.525	−2062.725	33.24	9.14	4.50
Tobit5	0.04828	0.07017	−1677.926	−1625.858	33.64	10	7.83
Tobit6	0.04830	0.06968	−1675.079	−1613.544	34.00	9.53	7.83
TPM2	0.04760	0.06881	−1878.537	−1850.744	34.19	9.52	6.33
TPM3	0.04746	0.06866	−1881.906	−1863.377	34.29	9.90	6.33
TPM5	0.04646	0.06811	−1893.829	−1858.611	33.81	9.72	4.58
**Beta5a(without)**	0.04612	0.06829	−2480.538	−2381.137	32.48	8.95	**2.00**
Beta2a(with)	0.04639	0.06887	−2550.758	−2489.224	31.91	9.72	2.92

The bold values indicates optimal model.

**Figure 2:**
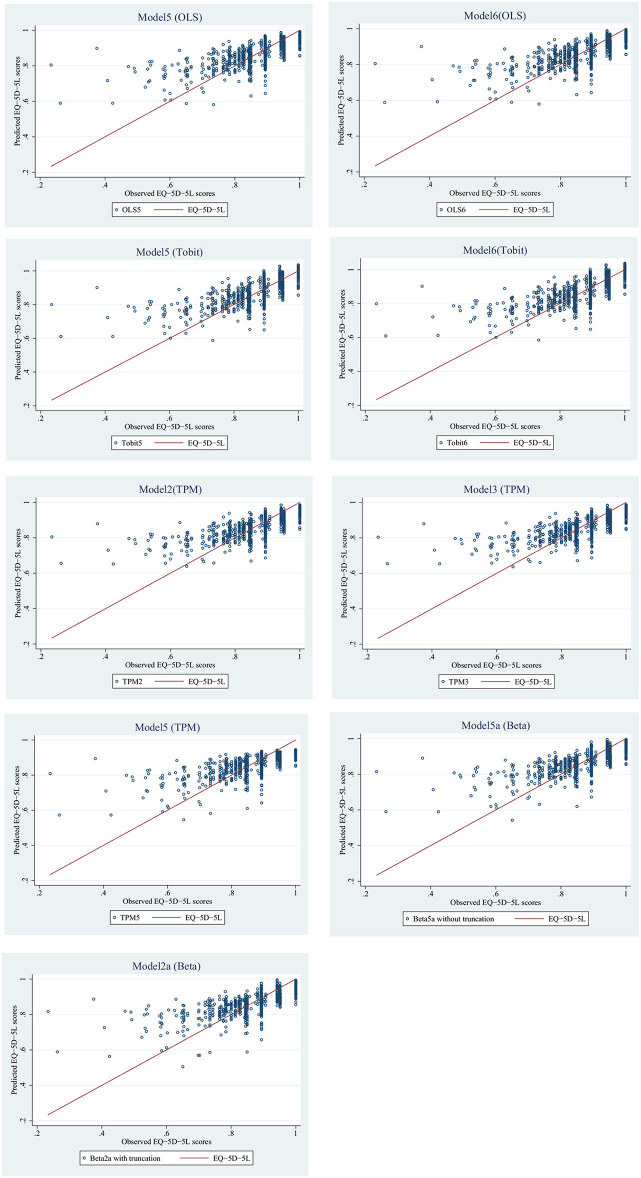
Correlation between observed and predicted utility values of nine optimal models.

**Figure 3:**
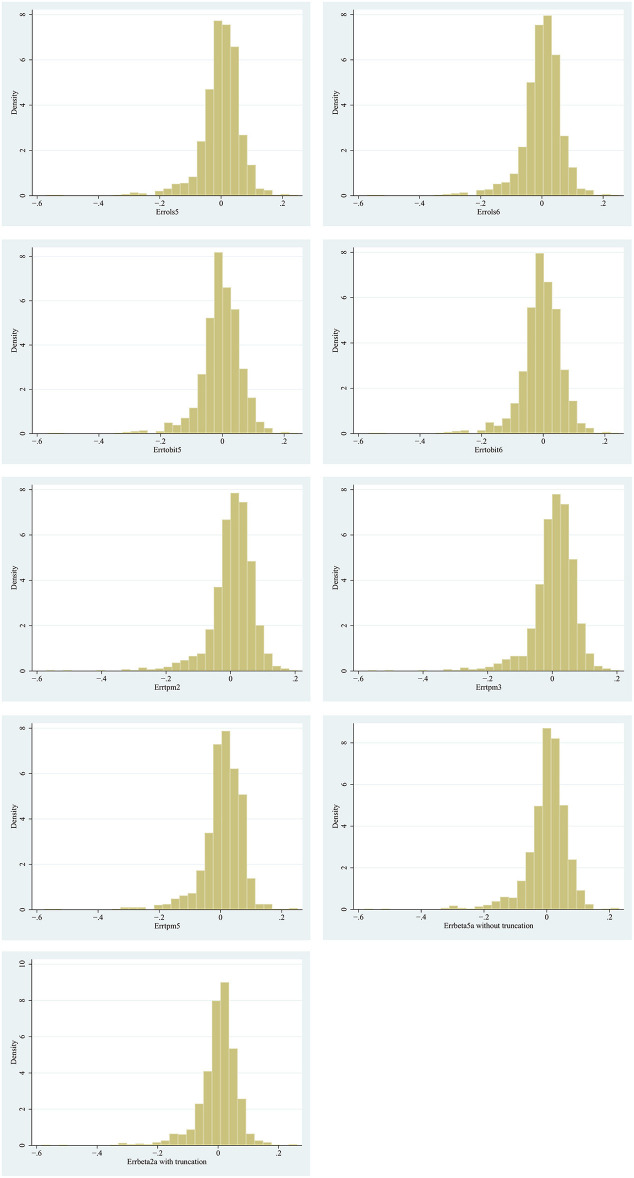
Predicting the error distribution of nine models.

**Figure 4:**
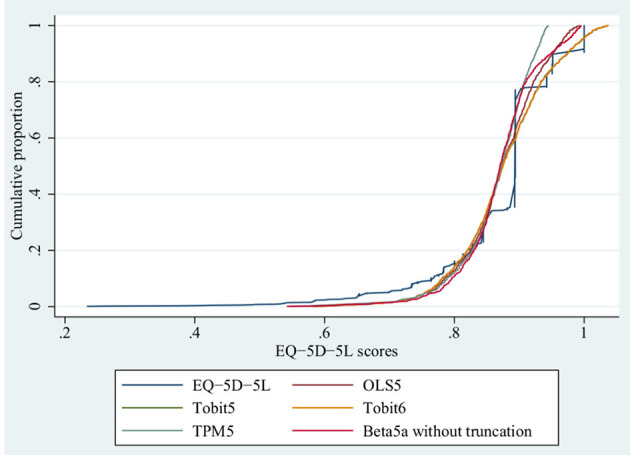
Estimation of conditional distribution function of EQ-5D-5L with the better models.

These nine models were used to estimate the predicted value of the EQ-5D-5L, and results were shown in Table 5, and their scatter plots and error histograms were plotted with these nine best models (Figures 2, 3). Based on the above results, OLS5, Tobit5, Tobit6, TPM5, and Beta5a (without truncation) were the best performing models in their respective mapping algorithms. The cumulative distribution function graph of these five models were shown below (Figure 4). Overall, the predicted value of Beta 5a (without truncation) was closest to the observed value. The excel calculator for the best-fitting model was provided in the Supplementary Table 5, which make it easy for the user to calculate the EQ-5D-5L from FACT-H&N scores.

**Table 5:** Predicted and observed values for EQ-5D-5L from the best-fitting model.

**Model**	**Mean**	**SD**	**Min**	**P10**	**P50**	**P90**	**Max**	**Upper bound(%)**
Observed values	0.87036	0.09393	0.23389	0.76408	0.89400	0.95168	1	0
OLS5	0.87036	0.06496	0.58135	0.78514	0.87403	0.95187	0.99262	0
OLS6	0.87036	0.06499	0.57909	0.78659	0.87450	0.95278	0.99548	0
Tobit5	0.87553	0.07270	0.58714	0.78119	0.87465	0.97340	1.03552	4.28571
Tobit6	0.87553	0.07271	0.58477	0.78175	0.87499	0.97446	1.03806	4.47619
TPM2	0.86523	0.05844	0.63902	0.78955	0.86873	0.93931	0.98620	0
TPM3	0.86523	0.05846	0.63686	0.78872	0.86880	0.94036	0.98678	0
TPM5	0.86327	0.05691	0.54648	0.78894	0.87242	0.92561	0.94507	0
Beta5a (without)	0.86939	0.06101	0.54193	0.79611	0.87054	0.94974	0.99510	0
Beta2a (with)	0.86967	0.06208	0.50488	0.79919	0.87333	0.94710	0.99619	0

## 4. Discussion

To knowledge, our study was the first to develop a mapping algorithm in PTC patients using the FACT-H&N and EQ-5D-5L and predict health utility values. We explored four different mapping methods with 32 models to find the most accurate predictive model to develop the mapping function. We mapped the FACT-H&N to the EQ-5D-5L using data from 1050 PTC patients in China.

EQ-5D itself has the nature of ceiling effect, resulting in utility scores that are not normally distributed (45). The EQ-5D-5L had lower rate in the ceiling effect than EQ-5D-3L (46, 47). In our study, a total of 101 (9.62%) respondents reported complete health, the ceiling effect of the EQ-5D-5L was not obvious, and the mean utility value of the EQ-5D-5L was 0.870 ± 0.094. Despite the mean EQ-5D-5L value was high, our ceiling effect was not evident, which may be due to the differences in health utility scores among thyroid patients with different status (surgery, Iodine 131, medicine treatment) in our study. It is worth noting that we also differed from other studies on EQ-5D-5L scores and ceiling effects. Compared with the other two breast cancer studies (48, 49), our EQ-5D-5L was higher (the other two: 0.5627, 0.52), and the ceiling effect was higher (the other two: 5.84%, 3.85%). This may be because different countries use different versions of the EQ-5D-5L, leading to differences in health preferences between countries. It could also be due to different sample sizes, diseases, and male to female ratios.

We found that the correlation coefficient between FACT-H&N and EQ-5D-5L was 0.621, indicating that there was a strong correlation between the two instruments. The variables included in the best model Beta 5a (without truncation point) were PWB, EWB, HNCS subscales scores and their squared and interactive terms scores, and the model was relatively robust. According to the five-fold cross-validation of the model, the Beta model had the lowest ARV, indicating that it fit the data best. According to the predicted value obtained by the model, the predicted value of Beta 5a (without truncation point) was nearest the observed value; thus, the Beta model was selected as the optimal model. Compared with several other mapping studies that used the Beta mixture regression model (40, 50, 51), our MAE, RMSE, AIC, BIC, and AE values were lower, and sample size was larger, which demonstrated that the model in our study was effective and achieved a better prediction performance.

Through extensive literature searches, we found that linear regression was typically used to map a quality of life instrument to the EQ-5D-5L, and the most commonly used model was OLS (37, 39, 40, 43, 52). Actually, the Beta model was more flexible because it was suitable for a bounded dependent variable in the interval and can take values at the boundary (40). In the mapping-related literature, the Beta mixture regression model has been used more in recent years (40, 50, 51). A systematic review noted that the introduction of square terms and interaction terms of independent variables into mapping models can improve model performance (53, 54). In our study, the OLS5, Tobit5, Tobit6, TPM5, and Beta 5a (without truncation) performed best, indicating that the square and interaction terms do improve model performance. Many studies illustrated that socio-demographic variables were independent varibles that affect the quality of life and can improve model performance (43, 52, 55). In the results of the study, we found gender to be statistically significant (*P* < 0.05) and did not find age to be statistically significant in predicting EQ-5D-5L scores. The significance of age found in other studies could be related to the type of cancer or the age range of the populations (56). According to the ISPOR guidelines (57), for most of mapping functions, the inclusion of age as a covariate was required even if not statistically significant. A recent systemic review of mapping studies showed that age was included as a potential predictor in 51% of the reported algorithms and gender was included in 55% (9). So we addded age and gender to Model 6. Only Tobit6 proved this, indicating that the improvement was small in our study.

The advantages of our study were as follows. First, the sample size was relatively large (*n* = 1,050). For other single-disease and single-center mapping-related studies, the sample size was primarily 200–300. Second, we used four mapping methods of direct mapping functions to develop a robust mapping model to predict the EQ-5D-5L health utility score in PTC patients, which can be used to assess the quality of life of PTC patients for use in health economics. Furthermore, to date, there were no published reports of the measurement of thyroid cancer health utility mapping models and related data on the Chinese population at home and abroad; thus, this study was important. Moreover, to improve the robustness of the model, this study performed five-fold cross-validation on the sample.

However, our study had the following limitations. First, due to the high survival rate and good prognosis of PTC patients, this cancer is commonly known as “happy cancer”, and the overall quality of life is better than that of other cancers. Respondents had generally high EQ-5D-5L scores, with only 24 (2.3%) having a utility value below 0.5, no negative utility value, and a small percentage of patients with poor quality of life, which may have affected the low-value prediction accuracy. Second, the sample size of the study was relatively large, but the survey was only conducted in one hospital and cannot be widely representative of all of China. Third, when examining the performance of mapping models, the lack of external validation in this study may limited the generalizability and extrapolation of its findings. Fourth, we used Chinese specific tariff, and whether the findings can be applied to other countries requires further research.

Despite these limitations, we demonstrated that the Beta mixture regression model performed much better than other models, and the mapping algorithm in the study could be used to predict the health utility value of the EQ-5D-5L in PTC patients.

## Data availability statement

The raw data supporting the conclusions of this article will be made available by the authors, without undue reservation.

## Ethics statement

The studies involving human participants were reviewed and approved by the Ethics Committee of Sichuan Cancer Hospital. The patients/participants provided their written informed consent to participate in this study.

## Author contributions

Data collection and analysis were performed by JP, NC, and LJ. The first draft of the manuscript was written by QY and DH. All authors contributed to the article and approved the submitted version.
