# Supplementary material for: Mapping study of papillary thyroid carcinoma in China: Predicting EQ-5D-5L utility values from FACT-H&N
Source: Front Public Health. 2023 Feb 23;11:1076879. doi: 10.3389/fpubh.2023.1076879 (PMC9998072; doi:10.3389/fpubh.2023.1076879)
Supplement: Supplementary file 1 [file Table_1.DOCX]

**Mapping study of papillary thyroid carcinoma in China: predicting EQ-5D-5L utility values from FACT-H&N**

**《Frontiers in Public Health》**

**Deyu Huang^1^, Jialing Peng^1^, Na Chen^1^, Qing Yang^2*^, Longlin Jiang^2^**

***Corresponding author**：**Qing Yang E-mail(s): [yangqingsc@163.com](mailto:yangqingsc@163.com)**

**No. 55, Section 4, Renmin South Road, Sichuan Cancer Hospital&Institute, Sichuan Cancer Center, School of Medicine, University of Electronic Science and Technology of China, Chengdu, 610041, China**

**Supplementary Table 1 Coefficient Estimation of OLS Models**

| **Variable** | OLS1 | OLS2 | OLS3 | OLS4 | OLS5 | OLS6 |
| --- | --- | --- | --- | --- | --- | --- |
| Constant term | 0.46243^***^ | 0.47633^***^ | 0.46441^***^ | 0.38947^***^ | 0.35279^***^ | 0.33902^***^ |
| FACT H&N total score | 0.00377^***^ |  |  |  |  |  |
| PWB |  | 0.01163^***^ | 0.01156^***^ | 0.02549^***^ | 0.03484^***^ | 0.03479^***^ |
| SWB |  | -0.00069 |  |  |  |  |
| EWB |  | 0.00338^***^ | 0.00335^***^ | -0.00021 | -0.00012 | 0.00032 |
| FWB |  | 0.00017 |  |  |  |  |
| HNCS |  | 0.00332^***^ | 0.00339^***^ | 0.00135 | -0.00344 | -0.00342 |
| **Square item of Dimension** |  |  |  |  |  |  |
| PWB squared |  |  |  | -0.00036^***^ | 0.00009 | 0.00008 |
| EWB squared |  |  |  | 0.00010 | 0.00042 | 0.00041 |
| HNCS squared |  |  |  | 0.00004 | 7.17e-06 | 5.53e-06 |
| **Interaction items for dimensions** |  |  |  |  |  |  |
| PWB×EWB |  |  |  |  | -0.00124^***^ | -0.00123^***^ |
| PWB×HNCS |  |  |  |  | -0.00013 | -0.00012 |
| EWB×HNCS |  |  |  |  | 0.00048^***^ | 0.00048^***^ |
| age |  |  |  |  |  | 0.00014 |
| gender |  |  |  |  |  | 0.00231 |

Note：^*^*P*＜0.10，^**^*P*＜0.05，^***^*P*＜0.01
