# Supplementary material for: Mapping study of papillary thyroid carcinoma in China: Predicting EQ-5D-5L utility values from FACT-H&N
Source: Front Public Health. 2023 Feb 23;11:1076879. doi: 10.3389/fpubh.2023.1076879 (PMC9998072; doi:10.3389/fpubh.2023.1076879)
Supplement: Supplementary file 2 [file Table_2.DOCX]

**Mapping study of papillary thyroid carcinoma in China: predicting EQ-5D-5L utility values from FACT-H&N**

**《Frontiers in Public Health》**

**Deyu Huang^1^, Jialing Peng^1^, Na Chen^1^, Qing Yang^2*^, Longlin Jiang^2^**

***Corresponding author**：**Qing Yang E-mail(s): [yangqingsc@163.com](mailto:yangqingsc@163.com)**

**No. 55, Section 4, Renmin South Road, Sichuan Cancer Hospital&Institute, Sichuan Cancer Center, School of Medicine, University of Electronic Science and Technology of China, Chengdu, 610041, China**

**Supplementary Table 2 Coefficient Estimation of Tobit Models**

| **Variable** | **Tobit1** | **Tobit2** | **Tobit3** | **Tobit4** | **Tobit5** | **Tobit6** |
| --- | --- | --- | --- | --- | --- | --- |
| Constant term | 0.41206^***^ | 0.43515^***^ | 0.42383^***^ | 0.49494^***^ | 0.48271^***^ | 0.47015^***^ |
| FACT H&N total score | 0.00429^***^ |  |  |  |  |  |
| PWB |  | 0.01256^***^ | 0.01261^***^ | 0.01835^***^ | 0.02756^***^ | 0.02757^***^ |
| SWB |  | -0.00060 |  |  |  |  |
| EWB |  | 0.00350^***^ | 0.00362^***^ | -0.00226 | -0.00241 | -0.00197 |
| FWB |  | 0.00051 |  |  |  |  |
| HNCS |  | 0.00389^***^ | 0.00403^***^ | -0.00165 | -0.00772^**^ | -0.00770^**^ |
| **Square item of Dimension** |  |  |  |  |  |  |
| PWB squared |  |  |  | -0.00015 | 0.00018 | 0.00018 |
| EWB squared |  |  |  | 0.00016 | 0.00040 | 0.00039 |
| HNCS squared |  |  |  | 0.00010^**^ | 0.00003 | 0.00003 |
| **Interaction items for dimensions** |  |  |  |  |  |  |
| PWB×EWB |  |  |  |  | -0.00116^***^ | -0.00115^***^ |
| PWB×HNCS |  |  |  |  | -0.00001 | -6.99e-06 |
| EWB×HNCS |  |  |  |  | 0.00054^***^ | 0.00053^***^ |
| age |  |  |  |  |  | 0.00014 |
| gender |  |  |  |  |  | 0.00133 |

Note：^*^*P*＜0.10，^**^*P*＜0.05，^***^*P*＜0.01
