# Supplementary material for: Mapping study of papillary thyroid carcinoma in China: Predicting EQ-5D-5L utility values from FACT-H&N
Source: Front Public Health. 2023 Feb 23;11:1076879. doi: 10.3389/fpubh.2023.1076879 (PMC9998072; doi:10.3389/fpubh.2023.1076879)
Supplement: Supplementary file 3 [file Table_3.DOCX]

**Mapping study of papillary thyroid carcinoma in China: predicting EQ-5D-5L utility values from FACT-H&N**

**《Frontiers in Public Health》**

**Deyu Huang^1^, Jialing Peng^1^, Na Chen^1^, Qing Yang^2*^, Longlin Jiang^2^**

***Corresponding author**：**Qing Yang E-mail(s): [yangqingsc@163.com](mailto:yangqingsc@163.com)**

**No. 55, Section 4, Renmin South Road, Sichuan Cancer Hospital&Institute, Sichuan Cancer Center, School of Medicine, University of Electronic Science and Technology of China, Chengdu, 610041, China**

**Supplementary Table 3 Coefficient Estimation of TPM Models**

| **Variable** | **TPM1** | | **TPM2** | | **TPM3** | | **TPM4** | | **TPM5** | | **TPM6** | |
| --- | --- | --- | --- | --- | --- | --- | --- | --- | --- | --- | --- | --- |
|  | First-part | Two-part | First-part | Two-part | First-part | Two-part | First-part | Two-part | First-part | Two-part | First-part | Two-part |
| Constant term | 4.69e-10^***^ | 0.50063^***^ | 2.03e-12^***^ | 0.50013^***^ | 1.29e-12^***^ | 0.49306^***^ | 1.25e-23^**^ | 0.30032^***^ | 4.84e-08 | 0.23491^***^ | 2.07e-08 | 0.21581^***^ |
| FACT H&N total score | 1.17519^***^ | 0.00336^***^ |  |  |  |  |  |  |  |  |  |  |
| PWB |  |  | 1.55473^***^ | 0.01124^***^ | 1.49425^***^ | 0.01120^***^ | 0.27760^**^ | 0.03139^***^ | 0.17787^*^ | 0.04108^***^ | 0.1716^*^ | 0.04097^***^ |
| SWB |  |  | 0.90201^**^ | -0.00040 |  |  |  |  |  |  |  |  |
| EWB |  |  | 1.24648^**^ | 0.00314^***^ | 1.22653^**^ | 0.00312^***^ | 2.14262 | 0.00087 | 0.52346 | 0.00132 | 0.55194 | 0.00186 |
| FWB |  |  | 1.02352 | 0.00008 |  |  |  |  |  |  |  |  |
| HNCS |  |  | 1.39941^***^ | 0.00260^***^ | 1.40024^***^ | 0.00263^***^ | 13.79909^**^ | 0.00482^*^ | 6.14366 | 0.00135 | 6.50500 | 0.00145 |
| **Square item of Dimension** |  |  |  |  |  |  |  |  |  |  |  |  |
| PWB squared |  |  |  |  |  |  | 1.03653^**^ | -0.00054^***^ | 1.01073 | 0.00006 | 1.00988 | 0.00005 |
| EWB squared |  |  |  |  |  |  | 0.98637 | 0.00006 | 0.97730 | 0.00048^*^ | 0.97618 | 0.00046^*^ |
| HNCS squared |  |  |  |  |  |  | 0.96751^*^ | -0.00004 | 0.94972^**^ | -0.00003 | 0.94868^**^ | -0.00003 |
| **Interaction items for dimensions** |  |  |  |  |  |  |  |  |  |  |  |  |
| PWB×EWB |  |  |  |  |  |  |  |  | 1.00246 | -0.00137^***^ | 1.00326 | -0.00135^***^ |
| PWB×HNCS |  |  |  |  |  |  |  |  | 1.04565 | -0.00026 | 1.04730 | -0.00026 |
| EWB×HNCS |  |  |  |  |  |  |  |  | 1.04943 | 0.00043^**^ | 1.04855 | 0.00043^**^ |
| age |  |  |  |  |  |  |  |  |  |  | 1.00258 | 0.00017 |
| gender |  |  |  |  |  |  |  |  |  |  | 0.85991 | 0.00386 |

Note：^*^*P*＜0.10，^**^*P*＜0.05，^***^*P*＜0.01
