# Supplementary material for: Mapping study of papillary thyroid carcinoma in China: Predicting EQ-5D-5L utility values from FACT-H&N
Source: Front Public Health. 2023 Feb 23;11:1076879. doi: 10.3389/fpubh.2023.1076879 (PMC9998072; doi:10.3389/fpubh.2023.1076879)
Supplement: Supplementary file 4 [file Table_4.DOCX]

**Mapping study of papillary thyroid carcinoma in China: predicting EQ-5D-5L utility values from FACT-H&N**

**《Frontiers in Public Health》**

**Deyu Huang^1^, Jialing Peng^1^, Na Chen^1^, Qing Yang^2*^, Longlin Jiang^2^**

***Corresponding author**：**Qing Yang E-mail(s): [yangqingsc@163.com](mailto:yangqingsc@163.com)**

**No. 55, Section 4, Renmin South Road, Sichuan Cancer Hospital&Institute, Sichuan Cancer Center, School of Medicine, University of Electronic Science and Technology of China, Chengdu, 610041, China**

**Supplementary Table 4 Coefficient Estimation of preferred Beta Models: Model 5a without truncation point**

| eq5d5l | Coefficient | Std. err. | z | P value | [95% conf. | interval] |
| --- | --- | --- | --- | --- | --- | --- |
| C1_mu |  |  |  |  |  |  |
| PWB | 0.141304*** | 0.033157 | 4.26 | 0.000 | 0.0763175 | 0.2062905 |
| EWB | -0.0351278 | 0.0418047 | -0.84 | 0.401 | -0.1170635 | 0.046808 |
| HNCS | -0.0078238 | 0.0182626 | -0.43 | 0.668 | -0.043618 | 0.0279703 |
| PWB squared | 0.0025519 | 0.0013094 | 1.95 | 0.051 | -0.0000145 | 0.0051183 |
| EWB squared | 0.0039552* | 0.0016381 | 2.41 | 0.016 | 0.0007446 | 0.0071657 |
| HNCS squared | -0.0002209 | 0.0003865 | -0.57 | 0.568 | -0.0009785 | 0.0005367 |
| PWB×EWB | -0.0083438*** | 0.0024117 | -3.46 | 0.001 | -0.0130707 | -0.003617 |
| PWB×HNCS | -0.0007243 | 0.001031 | -0.70 | 0.482 | -0.002745 | 0.0012964 |
| EWB×HNCS | 0.0029005** | 0.0010823 | 2.68 | 0.007 | 0.0007792 | 0.0050218 |
| _cons | -0.0357972 | 0.4294788 | -0.08 | 0.934 | -0.8775601 | 0 .8059658 |
| C1_lnphi |  |  |  |  |  |  |
| _cons | 3.925455*** | 0.0461504 | 85.06 | 0.000 | 3.835002 | 4.015908 |
| PM_ub |  |  |  |  |  |  |
| PWB | -1.726698 | 0.953153 | -1.81 | 0.070 | -3.594844 | 0.1414473 |
| EWB | -0.6472852 | 1.658906 | -0.39 | 0.696 | -3.898681 | 2.60411 |
| HNCS | 1.815422 | 1.242953 | 1.46 | 0.144 | -0.6207206 | 4.251564 |
| PWB squared | 0.0106749 | 0.0254387 | 0.42 | 0.675 | -0.0391841 | 0.0605339 |
| EWB squared | -0.0229571 | 0.0417193 | -0.55 | 0.582 | -0.1047254 | 0.0588111 |
| HNCS squared | -0.0515857* | 0.0216768 | -2.38 | 0.017 | -0.0940715 | -0.0090999 |
| PWB×EWB | 0.0024581 | 0.0511993 | 0.05 | 0.962 | -0.0978906 | 0.1028068 |
| PWB×HNCS | 0.0446425 | 0.0377978 | 1.18 | 0.238 | -0.0294399 | 0.1187249 |
| EWB×HNCS | 0.0482436 | 0.0387867 | 1.24 | 0.214 | -0.0277769 | 0.1242641 |
| _cons | -16.84292 | 25.24528 | -0.67 | 0.505 | -66.32276 | 32.63692 |
| C1_phi | 50.67612 | 2.338725 |  |  | 46.2935 | 55.47364 |

Note：* p<0.05, ** p<0.01, *** p<0.001
